# Virtual Simulation of the Effect of FMCW Laser Fuse Detector’s Component Performance Variability on Target Echo Characteristics under Smoke Interference

**DOI:** 10.3390/ma15124268

**Published:** 2022-06-16

**Authors:** Zhe Guo, Bing Yang, Yanbin Liang, Zhonghua Huang

**Affiliations:** School of Mechatronical Engineering, Beijing Institute of Technology, Beijing 100081, China; 3120185119@bit.edu.cn (Z.G.); 3120215164@bit.edu.cn (B.Y.); 3120195127@bit.edu.cn (Y.L.)

**Keywords:** FMCW, laser fuse, smoke backscattering, characteristic simulation, particle system, ray tracing, Unity3D

## Abstract

The laser transmitter and photoelectric receiver are the core modules of the detector in a laser proximity fuse, whose performance variability can affect the accuracy of target detection and identification. In particular, there is no study on the effect of detector’s component performance variability on frequency-modulated continuous-wave (FMCW) laser fuse under smoke interference. Therefore, based on the principles of particle dynamic collision, ray tracing, and laser detection, this paper builds a virtual simulation model of FMCW laser transmission with the professional particle system of Unity3D, and studies the effect of performance variability of laser fuse detector components on the target characteristics under smoke interference. Simulation results show that the difference in the performance of the fuse detector components causes the amplitude variation and peak migration of the beat signal spectrum, and the change in the visibility of the smoke can also affect the results, which indicates that the factors affecting the signal-to-noise ratio (SNR) of the echo signal are related to the smoke interference and performance variability of the detector. The proposed simulation model is supported by experimental results, which reflect the reliability of the proposed findings. Therefore, this study can be used for the optimization of the parameters in the laser fuse antismoke interference to avoid false alarms.

.

## 1. Introduction

As a control system for the terminal damage effectiveness of munitions, the laser proximity fuse is a triggerless optical fuse that uses a modulated laser beam to detect the target and detonate the weapon’s explosives, which has been widely used on various weapon models and platforms [1]. However, in aerosol environments such as smoke, laser fuse can be interfered with by suspended particles in the atmosphere at close range, causing misjudgment and false alarms; so, accomplishing accurate target detection in a smoky scene is one of the problems affecting a laser fuse to work properly under harsh surrounding conditions [2]. Compared with pulsed laser detection systems, the frequency-modulated continuous-wave (FMCW) laser is less affected by the time domain signal amplitude and echo waveform, and has the advantage of high range accuracy and anti-interference capability [3,4]. Under the smoke interference conditions, the laser beam will be affected by the backscattering of aerosol particles; so, it is very hard to carefully study the echo characteristics and extract the target distance information based on the actual measurement data of the differential frequency signal [5].

The main methods for studying the scattering process of FMCW lasers in smoke currently include experiment [6] and simulation [7,8]. In the case of the former method, it is more restricted by factors such as test situations and conditions, resulting in poor operability and repeatability of the method; so, it is generally used for comparative verification of results with other methods. For the simulation, especially the Monte-Carlo method, it is not constrained by the situation and only needs theoretical modeling and computer simulation, which can obtain reliable experimental data [9]. Therefore, the results of Monte-Carlo simulation have a close agreement with the real situation [10,11,12] and can effectively simulate multiple scattering of laser light in aerosol conditions [13,14], which has been widely used to study the target characteristics of FMCW laser in smoke environment [15,16].

However, the Monte-Carlo simulation method is used to simulate the complete motion process of photons in smoke by one-dimensional random sampling of the light range and scattering direction of photons [17]. The simulation process does not fully consider the influence of factors such as the physical properties of smoke particles [18], and ignores the three-dimensional dynamic collision and motion processes between particles. Moreover, it lacks the construction of the principal model of the virtual laser fuse, ignoring the simulation of the photon emission and reception processes of the internal detector. This cannot truly represent the three-dimensional dynamic collision and motion process of photons in the smoke particle scene. Therefore, the above single and static random motion process simulation is necessarily not able to fully reflect the complex, dynamic, nonrandom particle collision situation. In particular, the simulation results will show significant deviations and false alarms in the low-visibility smoke scene. At the same time, a large number of photon simulations and parameter sampling can reduce the efficiency of simulation [19].

The root cause for the above problem is that the traditional simulation method can only perform low-dimensional and superficial characterization of photon transport simulation in smoke, and it is not efficient to achieve simultaneous cosimulation of multiple systems among the smoke system, laser transmitting system, and laser receiving system. In order to solve this problem, it is possible to integrate the advantages of virtual reality technology and traditional simulation methods with each other based on the validity and reliability of deep simulation with particle systems in a virtual reality approach [20,21]. As a result, based on laser detection and Monte-Carlo principles, the three-dimensional dynamic and virtual simulation model that meets the requirements of simultaneous multisystem cosimulation can be built using a professional and virtual particle system, such as in Unity3D [22]. In this way, not only can the deep correlation between the theoretical model and the real simulation be realized, but the simulation method can also be transformed to a higher dimensional and deeper level, which can significantly improve the reliability of the simulation results of echo signals.

This paper is organized as follows. Section 2 introduces the FMCW laser detection principle and the echo signal model. Section 3 describes the principle analysis of laser echo signal simulation based on the 3D particle system in a smoke-free environment. Section 4 describes the principle analysis of laser echo signal simulation based on ray tracing in smoke environment. Section 5 simulates and analyses the effects of three types of laser fuse component differential factors on the target echo characteristics using the dynamic particle collision-based laser echo characteristics simulation method, respectively. Section 6 provides validity experiment of the proposed simulation method. Section 7 summarizes the work of this paper.

## 2. FMCW Laser Backscattering Signal Model

**Figure 1:**
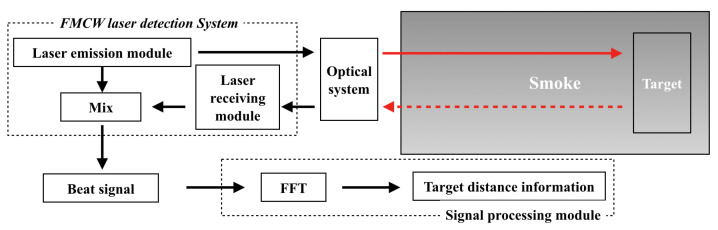
Block diagram of FMCW laser fuse detection process.

The block diagram of the FMCW laser detection principle is shown in Figure 1 [23]. The laser transmitter module divides the linear frequency-modulated (FM) signal into two parts: one part of FM signal is used to modulate the laser light intensity, which is collimated by the optical system and launched into the smoke to detect the target; the other part of FM signal is used as the local oscillation signal, and the laser echo signal received by the receiver module is mixed and low-pass-filtered to output a beat signal, which is processed by the signal processing module to achieve the analysis of target characteristics and the extraction of information such as distance and speed of the target. In signal processing module, Fast Fourier Transform (FFT) is an efficient and fast computational method for Discrete Fourier Transform (DFT) in the field of signal processing, which can be combined with high-speed hardware to achieve real-time processing of signals.

According to the FMCW laser detection principle, the values of the transmitted signal $S_{T}\left(t\right)$ and the instantaneous frequency $f_{T}\left(t\right)$ of the laser detection system based on the triangular wave FM method at a single FM period can be expressed as

(1) $$S_{T}\left(t\right)=P_{0}+P_{T}cos\left(2\pi f_{0}t+2\pi \left(\frac{B}{T_{m}}t^{2}\right)+\varphi_{0}\right),t\in \left[0,T_{m}\right],$$

(2) $$f_{T}\left(t\right)=f_{0}+2\frac{B}{T_{m}}t,t\in \left[0,T_{m}\right],$$

where $P_{0}$ and $P_{T}$ represent the DC and AC components of the laser power, $f_{0}$ is the initial frequency of the FM signal, *B* is the FM bandwidth, $T_{m}$ is the modulation period, and $\varphi_{0}$ is the initial phase.

**Figure 2:**
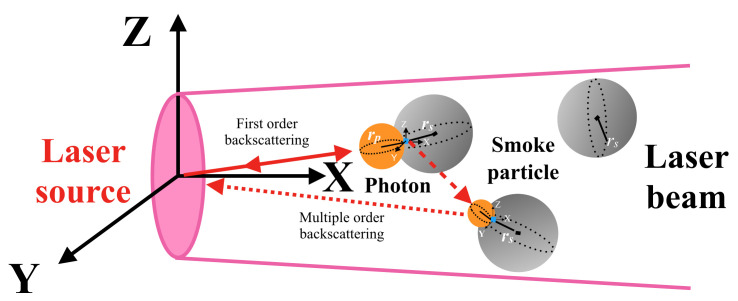
Collision and scattering process of photons and smoke particles.

**Figure 3:**
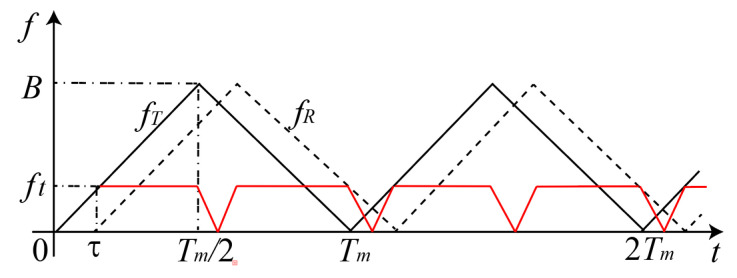
FMCW detection principle based on triangle wave frequency modulation.

During laser transmission in a smoky environment, the motion of photons is divided into two main types [8]: (1) They are received by the detector after being reflected from the target surface. (2) They are received by the detector after being backscattered by the smoke particles several times, and the schematic diagram of the collision scattering process is shown in Figure 2. Therefore, the laser echo signal $S_{R}\left(t\right)$ can be expressed as

(3) $$S_{R}\left(t\right)=k_{t}\left(t\right)\cdot S_{T}\left(t-\tau_{t}\right)+k_{t}\left(t\right)\cdot S_{T}\left(t-\tau_{s}\right)+k_{n}\left(t\right)\cdot S_{n}\left(t\right),$$

where $k_{t}\left(t\right)$ and $k_{s}\left(t\right)$ are the target reflectance and transmission attenuation coefficient, respectively; $\tau_{t}$ and $\tau_{t}$ are the echo signal time delays of the two processes, respectively; and $S_{n}\left(t\right)$ is the random noise signal. The beat signal $S_{B}\left(t\right)$ is obtained by mixing $S_{T}\left(t\right)$ with $S_{R}\left(t\right)$ and low-pass filtering; then, it is expressed as

(4) $$\begin{array}{c} S_{B}\left(t\right)=\frac{1}{2}k_{t}\left(t\right)\cdot P_{T}\left(t\right)cos\left(2\pi f_{t}t+\varphi_{t}\right)+\frac{1}{2}k_{s}\left(t\right)\cdot P_{T}\left(t\right)cos\left(2\pi f_{s}t+\varphi_{s}\right)+k_{n}\left(t\right), \end{array}$$

where $f_{t}$ and $f_{s}$ denote the beat frequency generated by the target echo and smoke scattering signal, respectively; $\phi_{t}$ and $\phi_{s}$ denote the phase delay between the target echo signal and smoke scattering signal due to mixing, respectively. According to the frequency shift property of the Fourier transform, the power spectral density expression of the beat signal is expressed as

(5) $$|S_{B}\left(t\right)|=\frac{1}{4}|K_{t}(f-f_{t})|+\frac{1}{4}|K_{s}(f-f_{s})|+|K_{n}\left(f\right)|,$$

where $K_{t}\left(f\right)$ and $K_{s}\left(f\right)$ are the results of the fast Fourier transform (FFT) of $k_{t}\left(t\right)$ and $k_{s}\left(t\right)$, respectively, corresponding to the amplitude of target echo signal and smoke scattering signal spectrum. According to Equation (2) and Figure 3, it can be seen that the beat frequency rate $f_{t}=k\tau$, the sweep slope $k=\frac{2B}{T_{m}}$, and the laser transmission time $\tau =\frac{2R}{c}$. $f_{R}$ is expressed as the received frequency and shown as the dashed line in Figure 3. The target distance can be calculated as [24]

(6) $$R=\frac{cT_{m}}{4B}f_{t},$$

where *c* and *R* are the speed of light and target distance, respectively. It can be seen that the relationship between beat frequency and target distance is a bijection based on Equation (6).

## 3. Simulation Process Analysis of Laser Echo Signal Based on 3D Particle System in Smoke-Free Environment

In order to obtain reliable laser echo characteristics in a smoky environment, it is necessary to obtain reliable laser target echo data under smoke-free conditions and to accurately calculate the actual distance of the target based on the beat frequency signal. According to the FMCW laser detection principle of Section 1, the simulation process of laser echo signal in smoke-free environment needs to completely simulate the spatial motion process of photons, which mainly includes photon emission process simulation, flight simulation, and reception simulation. Since the optical intensity of the FMCW laser is obtained by modulation of the FM signal, the instantaneous optical power of the modulated FMCW laser can be simulated by the number of emitted photons [15]. Therefore, based on the principle of laser detection and Monte Carlo simulation, the FMCW laser echo signal simulation process can be generally described as follows [15,16]: (1) The number of photons emitted at each moment is determined according to the laser power emission signal amplitude; in the range of the divergence angle of the laser source, photons are sequentially emitted to the smoke scene in time order. (2) The final laser echo signal can be obtained by counting the energy and flight time of all photons from the time of emission to the reception.

**Figure 4:**
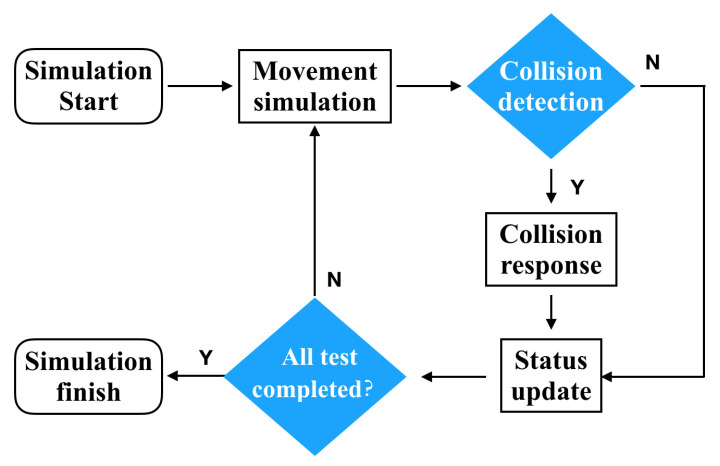
Flow chart of particle system dynamic simulations.

For simulating realistic behavior in realistic scenes, particle systems are able to effectively merge mathematical complexity with model accuracy. Therefore, they are some of the most commonly used models in 3D dynamic simulations based on physical modeling, and currently, the most popular model for multidomain dynamic simulation. As the core unit in the particle system, the particle’s mass is usually concentrated in centroid, which not only greatly simplifies the dynamics equations of particles, but also reduces the complexity of solving the particle dynamics equations by applying all the interaction forces between the particles to the centroids, effectively improving the speed of simulation [25,26]. During the particle system simulation, all external forces applied to the particle are calculated and integrated by counting the information of particle collisions during the simulation. Then, the states at the initial and end simulation moments of the particle are used as the upper and lower bounds of the integration condition, respectively, and the motion state of the particle can be integrated numerically. In this way, basic dynamic simulation runs of particle systems can be implemented. The flow chart of the particle system dynamics simulation is shown in Figure 4.

**Figure 5:**
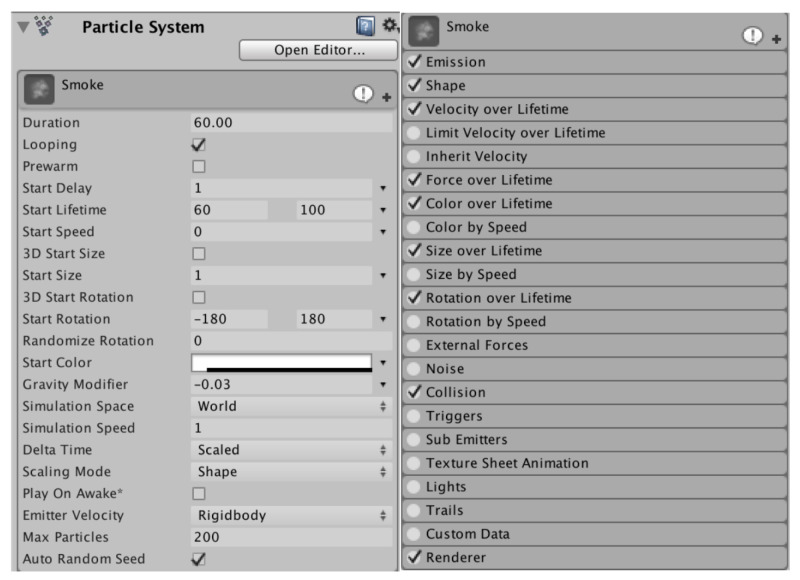
Particle system modules in Unity3D.

Moreover, in order to improve the simulation efficiency, the simulation process should avoid solving a large number of complex kinetic equations. Based on the ability to adequately simulate the behavior of FMCW laser detection systems, it is possible to simulate photon motion processes using specialized particle systems with physical properties. As a major implementation of virtual reality, Unity3D can make the simulation highly interactive and realistic in 3D based on internal physics and optics engines, which provide conditions for scientific simulation. In particular, based on these engines, Unity3D’s professional particle system includes emitters, animations, and renderers for simulation, which can create unique particle systems to meet the needs of smoke scenes. Thus, this paper uses the professional particle system in Unity3D physics engine to build a realistic virtual environment that meets the test environment and various conditions [27]. The parameters of the particle system in Unity3D engine, such as the attribute module, emission module, shape module, and force and collision module, can be seen in Figure 5.

**Figure 6:**
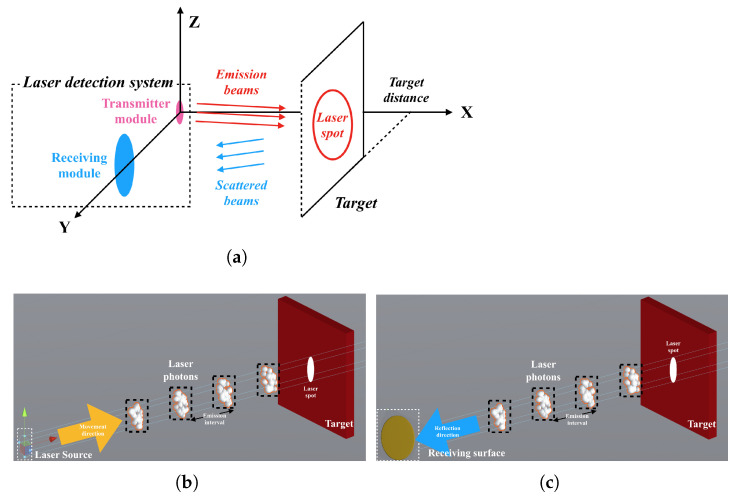
FMCW laser simulation process based on particle system in Unity3D: (**a**) laser detection process, (**b**) emission process, (**c**) receiving process.

**Figure 7:**
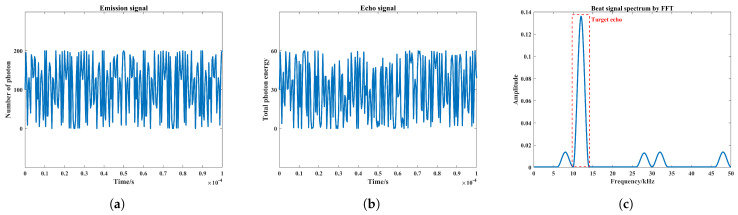
Laser emission signal, echo signal at a target distance of 3 m and beat signal spectrum: (**a**) Laser emission signal, (**b**) echo signal, (**c**) beat signal spectrum.

According to the FMCW laser detection process in the smoke-free environment, as shown in Figure 6a, the photon emission process and reception process are simulated in a Unity3D virtual scene with the professional particle system, respectively. As an example, the distance between the laser detection system and the rigid target is set to 3 m, the reflectivity of the rigid-body target is set to 0.3, and the laser beam divergence angle is set to 5°; the virtual simulation process can be seen in Figure 6b,c. The sweep bandwidth *B*, period $T_{m}$, and slope *k* are set to 100 MHz, 0.5 ms, and 600 GHz/s, respectively, and the corresponding FMCW laser power emission signal model can be seen in Figure 7a. The receiving field of view (FOV) is set to 45°, and the echo signal results at this time are shown in Figure 7b. Thus, the beat frequency signal is obtained by mixing the laser emission signal with the echo signal; then, the spectrum results can be obtained after FFT processing, as shown in Figure 7c.

In particular, considering that only preset targets exist in the smoke-free scene, the spectrum peaks in Figure 7c also only represent the target echoes. At this time, the beat frequency corresponding to the peak of the spectrum $f_{b}$ is 12 kHz, and the detection distance *D* can be calculated as 3 m according to Equation (7), which is consistent with the actual distance of the preset target. Therefore, it can be considered here that the simulation of echo signal based on 3D particle system is feasible and effective, and the simulation results are reliable enough, which provides the basis for the subsequent simulation of echo characteristics in smoke scenes.

(7) $$D=\frac{T_{m}}{2B}\cdot \frac{c}{2}\cdot f_{b}\approx \frac{3\times 10^{8}\;m/s}{2\times 600\;\mathrm{GHz}/s}\times 12\;\mathrm{KHz}=3\;m$$

## 4. Simulation Process Analysis of Laser Echo Signal Based on Ray-Tracing Principle in Smoke Environment

In the smoke environment, the simulation process of laser echo signal needs not only to completely simulate spatial motion process of photons but also to reflect various physical collision processes of photons in the motion process, especially the collision process of photons with smoke particles and the collision process of photons with rigid body targets. According to the simplifying property of the particle system to particle dynamics equation of Section 3, the dynamic simulation model based on particle system can effectively simulate the motion and collision process of photons. Therefore, it is only necessary to replace the smoke-free environment with smoke environment, and the simulation process of Section 3 can be followed to simulate the echo signal in a smoke environment. However, as the number of photons emitted from the light source is extremely large, only a few photons are received through scattering or reflection between the surfaces of the objects in the scene and there is bound to be a large number of wasted photons in the simulation process, which affects the operation efficiency of the simulation. If the number of photons emitted is reduced, it will inevitably reduce the number of photons received after reflection by the target, thus causing a reduction in the amplitude of target echo. Therefore, the factor affects the reliability of simulation results.

**Figure 8:**
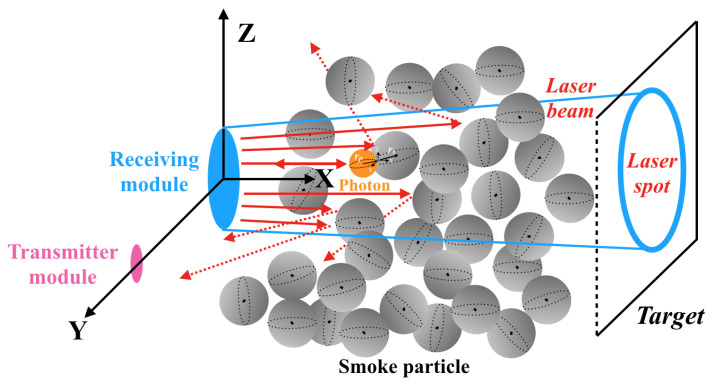
Photon motion process based on reverse ray-tracing.

In order to improve the authenticity of the simulation and also to reduce the emission of useless photons, this paper is based on the reverse ray-tracing [28] approach to simulate the echo signal of the laser beam emitted outward from the receiver module based on the receiving field-of-view angle, and the simulation process is shown in Figure 8. The simulation process can be described as follows: according to the ray-tracing algorithm, the laser beam emitted from the receiver is decomposed into several independent rays represented by photons, and each photon is tracked in the smoke particle environment until it is received by the transmitter. Moreover, for an isotropic monochromatic light source, each ray needs to carry a similar power, so that all emitted rays are distributed as evenly as possible.

In the transmission of laser in smoke environment, there will be a large number of smoke particles with random locations due to the smoke scene, and multiple collisions between the laser photons and smoke particles may happen at different locations. The received echo signal is necessarily a mixture of multiple types of smoke echoes and target echoes [8]. Based on the Fourier transform principle, any waveform can be represented by a linear superposition of multiple sinusoidal functions, where each component function has a corresponding frequency, phase, and amplitude [29]. The laser echo signal simulation can be transformed into the process of obtaining the smoke echo signal and the target echo signal separately and then superimposed.

During the process of emitting photons outward along each individual ray at the receiving module, it is assumed that all linear distances between the initial position of the photon and smoke particle or target surface can be detected by Unity3D as $l_{i}$, so that all laser echo signals at different distances can be obtained. The time and amplitude of these signals are, respectively, used as the number of emissions and photons for the photon emission model of the receiving module, and the simulation is performed by emitting photons outward from the receiver.

Considering that the premise of reverse ray-tracing simulation is that the receiver module has received all the photons emitted from the transmitter and meets the reception conditions at each moment, it can be assumed that the expression of the corresponding echo signal at each moment is known as Equation (8) based on Equation (3), and the purpose of the simulation is to determine the parameter values of the final laser echo signal.

(8) $$S_{R_{i}}\left(t\right)=k_{i}\left(t\right)\cdot \left[P_{0}+P_{T}cos\left(2\pi f_{0}\left(t-\frac{2l_{i}}{c}\right)+\frac{2\pi B}{T_{m}}{\left(t-\frac{2l_{i}}{c}\right)}^{2}+\varphi_{0}\right)\right]$$

Thus, the simulation process only needs to calculate the total photon energy at each emission moment when the photons are emitted from the receiver module until they are received by the transmitter module. In this way, we only need to record the photon energy to obtain the laser echo signal, and it is not necessary to record the flight time of photons in the smoke scene. The final expression of the laser echo signal is shown in Equation (9).

(9) $$S_{R}\left(t\right)=\sum_{i=1}^{i_{max}}k_{i}\left(t\right)\cdot S_{T}\left(t-\frac{2l_{i}}{c}\right)$$

In the transmission process of photons, the collisional motion process of photons can be divided into the collision process with the target surface and the collision process with smoke particles. The direction of motion of photons after collision is determined by force analysis at the collision cross section, and the energy after collision is calculated according to the target reflectivity. When the photon falls into the receiving area, it can be considered that the photon is received by the detector. The photon energy *E* at this point can be calculated as [15]

(10) $$E_{n_{r}+n_{s}}={\left(r_{t}\right)}^{n_{r}}\cdot {\left(r_{s}\right)}^{n_{s}}\cdot r_{a}\cdot E,$$

where $n_{r}$ and $n_{s}$ are the number of photon collisions with the target and the number of collisions with smoke particles, respectively; $r_{t}$, $r_{s}$, and $r_{a}$ are the coefficient of target reflectivity, scattered energy loss, and atmospheric attenuation coefficient, respectively. $r_{s}$ and $r_{a}$ can be calculated by [16,30]

(11) $$r_{s}=\frac{\gamma_{sca(x,m)}}{\gamma_{ext(x,m)}},$$

(12) $$r_{a}=e^{-\sigma L},$$

where the smoke particle size parameter is $x=\frac{2\pi r}{\lambda }$, *r* is smoke particle radius, λ is laser wavelength, the particle complex refractive index is $m=m_{1}-im_{2}$, σ is atmospheric attenuation coefficient, *L* is photon transmission distance; scattering coefficient $\gamma_{sca(x,m)}$ and extinction coefficient $\gamma_{ext(x,m)}$ are calculated as [31]

(13) $$\gamma_{sca(x,m)}=\pi \int_{r_{min}}^{r_{max}}Q_{sca}\left(x,m\right)\cdot n\left(r\right)\cdot r^{2}dr,$$

(14) $$\gamma_{ext(x,m)}=\pi \int_{r_{min}}^{r_{max}}Q_{ext}\left(x,m\right)\cdot n\left(r\right)\cdot r^{2}dr,$$

where *n*(*r*) denotes the particle size distribution of the smoke particles, $Q_{sca}\left(x,m\right)$ and $Q_{sca}\left(x,m\right)$ are scattering and extinction factors, respectively. The photon energy attenuation is influenced by the scattering factor $Q_{sca}$ and the extinction factor $Q_{ext}$, whose approximated formula is calculated as [32]

(15) $$\left\{\begin{array}{cc} Q_{sca}= & 1+\frac{e^{-4xm_{2}}}{2xm_{2}}+\frac{e^{-4xm_{2}}-1}{8x^{2}m_{2}^{2}}\\ Q_{ext}= & 2+4{\left(\frac{cos\theta }{\rho }\right)}^{2}cos2\theta -4e^{-\rho tan\theta }\cdot \left[\frac{cos\theta }{\rho }sin\left(\rho -\theta \right)-{\left(\frac{cos\theta }{\rho }\right)}^{2}cos\left(\rho -\theta \right)\right] \end{array}\right.$$

where ρ=2x(m−1) and $tan\theta =\frac{m_{2}}{m_{1}-1}$. Therefore, during each launch moment, all the photon energies received at the transmitter can be calculated according to Equation (10), and the laser echo signal can be obtained. Then, the echo signal is mixed with the transmit signal and low-pass filtered to obtain the beat signal. After the FFT processing, it can extract the target distance information from the spectrum of beat signal.

## 5. Simulation of Laser Echo Characteristics Based on Dynamic Particle Collisions

The actual performance parameters of the laser diode and photodiode, which are the core components of the transmitter and receiver of the fuse prototype [33], are usually inconsistent with the factory specifications, and the performance loss is unavoidable in the process of actual use. Therefore, considering the uncertainties in the performance and lifetime of the laser diode at the transmitter and the photodiode at the receiver of the laser fuse prototype, the error factors of these prototypes should be incorporated into the characteristic simulation, such as laser source emission power error, laser source divergence angle error, and receiving FOV error. Only by analyzing the influence of these error factors on the echo characteristics results can we ensure that the simulation results obtained are more authentic and reliable.

### 5.1. Simulation Steps and Results

In the actual environment, as smoke is an aggregate with multiple particle sizes, the particle size is mainly distributed between a few microns to tens of microns, and the subsequent study of the effect of particle size on laser fuse detection performance is not meaningful [34]. So, the particle size of the smoke in this simulation is set to the same value, and sets the smoke scene only by controlling the smoke concentration. In particular, considering that smoke visibility can be used to characterize the smoke concentration, according to the expression of smoke visibility and smoke concentration [35]:

(16) $$V=\sqrt[{y}]{\frac{c_{0}}{m_{c}}},$$

where $m_{c}$ is smoke concentration in $g/m^{3}$; *V* is smoke visibility in meters; $c_{0}$ and *y* are constants that depend on the type of smoke environment and their values are 37.3 and 1.07, respectively. In this way, the correspondence between smoke visibility and concentration can be obtained; thus, the number of simulated smoke particles at different smoke visibility can also be determined. In order to reflect the complexity and uncertainty of the smoke, the spatial position of smoke particles can be set by a random distribution. Therefore, based on the simulation analysis in smoke-free and smoke environment from Section 3 and Section 4, the complete flow of proposed simulation method can be described as

1. Determine the smoke visibility and build the corresponding virtual smoke particle scene;
2. According to the preset distance information between the laser source and the target, set up the virtual laser detection system and the target to be measured in the scene, respectively;
3. Perform ray collision detection according to FOV of the receiver, obtain all the linear distances $l_{i}$ between the receiver and the smoke particles or the target, and determine all the photon emission models based on the FMCW laser echo signal expression;
4. Based on the photon emission model, the receiving module fires the corresponding number of spherical photons to the smoke particle scene in sequence with the initial moment, and records the spatial position information of the current photons;
5. When the photons collide with the smoke particles, record the photon position and update the flight path, and calculate the current photon energy after the collision by Equation (10); When the photons collide with the target, record the photon position and flight path, and calculate the current photon energy based on the target reflectivity;
6. After the photon flies out of the smoke scene, calculate all the photon energy received by the transmitting module at the current moment. According to the moment order to launch photons, count the total photon energy at each moment, and obtain the laser echo signal;
7. Repeat Steps 4–6 in this flow for all photon emission models and superimpose the corresponding laser echo signals to obtain the final FMCW laser echo signal. The beat signal and spectrum for extracting the distance information can be obtained after frequency mixing and FFT processing.

**Figure 9:**
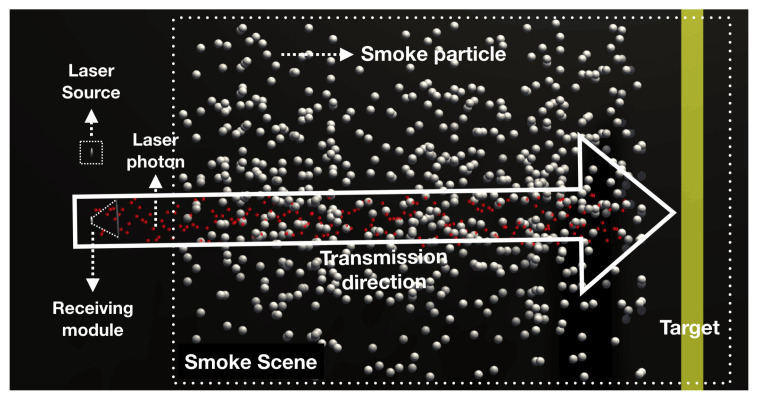
Photon motion process based on reverse ray-tracing in Unity3D.

**Figure 10:**
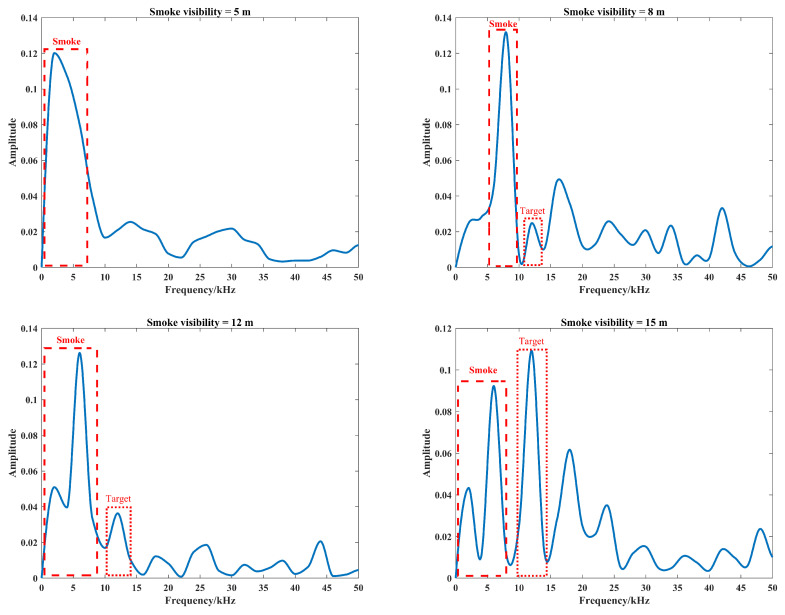
Spectrum of beat signal by proposed method at different smoke visibilities.

In the virtual smoke scene, the distance between the target and the laser detector is set to 3 m. Based on the theoretical model and simulation model of photon transmission process, the echoes are simulated under the conditions of 5 m, 8 m, 12 m, and 15 m of smoke visibility, and the atmospheric attenuation coefficient can be set to $\sigma =\frac{3.912}{V}$ by [36]. The main parameters of the echo signal simulation are shown in Section 3, the smoke test scene and test flow diagram built in the Unity environment are shown in Figure 9, and the simulation results of echo signal spectrum are shown in Figure 10.

As can be seen in Figure 10, the echo signal contains smoke echoes and target echoes, and the scattering of smoke particles at low visibility can cause multiple interfering echoes, when the target echoes are drowned in the smoke noise. The mixing of smoke echoes and target echoes causes an expansion of the beat frequency signal spectrum, which is consistent with the conclusion from [15]. Considering that the function of the signal-receiving module is to detect the frequency corresponding to the peak of the beat frequency signal spectrum, the signal-to-noise ratio (SNR) of the beat frequency signal in smoke can be defined as shown in Equation (17), where $f_{t}$ and $f_{s}$ are the frequencies corresponding to peak of target echo and smoke echo, respectively. With the increase in smoke visibility, the amplitude of the target echo will gradually increase, and the SNR of beat signal spectrum will also gradually increase. According to the calculation result from Equation (7), the target distance can be calculated directly from the frequency corresponding to the extreme point of beat frequency spectrum at the visibility of 15 m.

(17) $$SNR=10log_{10}\frac{K_{f}\left(f_{t}\right)}{K_{s}\left(f_{s}\right)}$$

### 5.2. Effect of Laser Source Emission Power Error on Echo Characteristics

**Figure 11:**
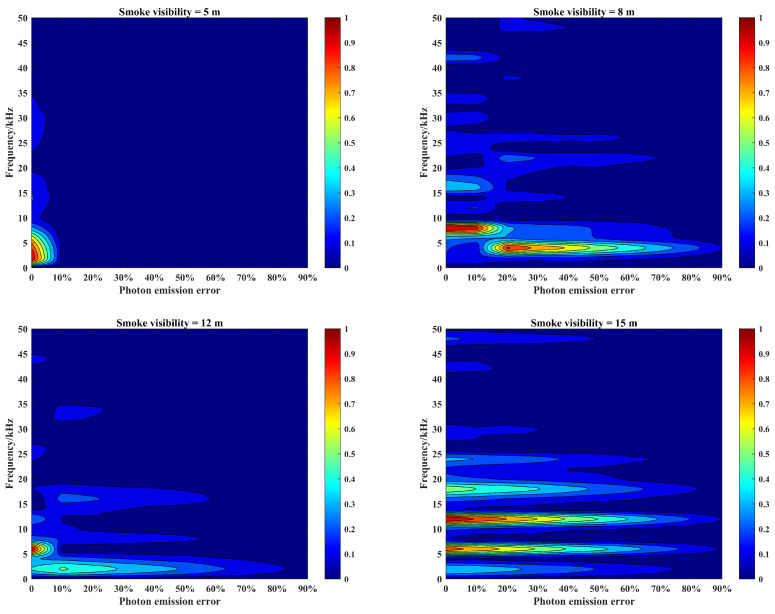
Amplitude–frequency characteristics of beat signals corresponding to photon emission errors of laser source under different smoke visibility conditions.

Under different smoke visibility conditions, according to the above echo signal simulation process, in order to describe the effect of laser emission power difference on the echo characteristics, it is necessary to simulate the effect of photon emission error on the target echo characteristics. The simulation parameters are still the same as those in Section 3, and the number of photon emission errors is set to 0–90% of the initial value under the smoke visibility conditions of 5 m, 8 m, 12 m, and 15 m. The amplitude and frequency characteristics of beat signals are shown in Figure 11.

As can be seen in Figure 11, the laser source emission power error will reduce the laser emission energy and decrease the laser penetration ability in the smoke, which will have an impact on the echo characteristics: (1) The peak migration will appear in the beat signal spectrum. In lower smoke visibility conditions, such as smoke visibility of 8 m, the photon emission error will not only cause a reduction in the beat signal spectrum amplitude, but also make the spectrum peak migrate to a lower frequency band. (2) The peak migration of the beat signal spectrum is affected by the change of smoke visibility. With the increase in smoke visibility from 8 m to 12 m, the trend of spectrum amplitude relatively slows down, resulting in the peak migration caused by the photon emission error value of 20% to change to 10%. At this time, the peak migration will gradually disappear, but the photon emission error will be more sensitive to it. (3) Peak migration only occurs in the low visibility of smoke conditions. In higher smoke visibility conditions, such as smoke visibility increased to 15 m, the impact of photon emission error on the target echo characteristics is only reflected in the change in amplitude, the spectral peak migration no longer appears. (4) Only the smoke echo signal will present the peak migration of the amplitude and frequency. When the peak migration appears, the target echo signal amplitude will be greatly reduced. At the same time, the SNR of the beat signal spectrum will be greatly reduced, and the target echo signal cannot be extracted from the echo signal at this time, which is obviously not beneficial to target detection and identification.

### 5.3. Effect of Laser Source Divergence Angle Error on Echo Characteristics

**Figure 12:**
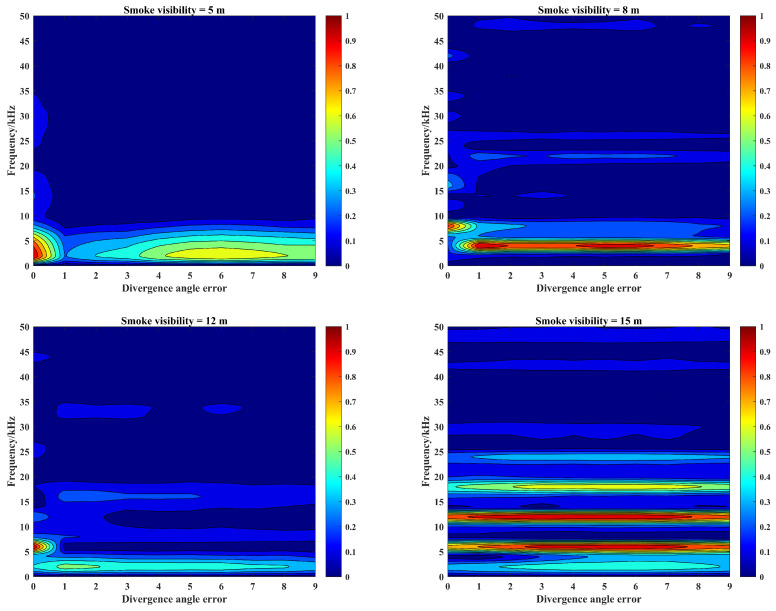
Amplitude–frequency characteristics of beat signals corresponding to the field of view errors of photoelectric detector under different smoke visibility conditions.

Under different smoke visibility conditions, according to the above echo signal simulation process, in order to describe the effect of laser divergence angle difference on the echo characteristics, it is necessary to simulate the effect of divergence angle error on the target echo characteristics. The simulation parameters are still the same as those in Section 3, and the number of divergence angle error is set to 0–9 times of the initial value under the smoke visibility conditions of 5 m, 8 m, 12 m, and 15 m. The amplitude and frequency characteristics of beat signals are shown in Figure 12.

In Figure 12, it can be seen that the laser source divergence angle error will reduce the laser collimation effect, increase the irradiation area of the light source and enhance the effect of smoke particle backscattering. Meanwhile, this can cause the energy dispersion of laser source, and decrease the penetration ability of the laser in the smoke, which will have an impact on the echo characteristics: (1) The peak migration will appear in the beat signal spectrum. The laser source divergence angle error will not only cause the beat signal spectrum amplitude reduction, also make the spectrum peak migration to lower frequency band. (2) The peak migration of the beat signal spectrum is not affected by the change of smoke visibility. With the increase in smoke visibility, the amplitude of the beat signal spectrum will change, but the thresholds of peak migration always remain the same. (3) The effect of divergence angle error on the peak migration is related to the smoke visibility. In low-visibility smoke scenes, a small divergence angle error will cause peak migration; however, in high-visibility smoke scenes, only a large divergence angle error can cause peak migration. (4) The effect of divergence angle error on the SNR of echo signal is related to the smoke visibility. When the peak migration appears, the target echo signal amplitude under the smoke visibility of 8 m will be significantly reduced, and the SNR of the beat signal spectrum will be also reduced at this time. However, the target echo signal amplitude under the smoke visibility of 12 m has no significant change. Considering the reduction of the smoke echo signal amplitude, the SNR will increase at this time and the target echo signal can be extracted from the echo signal.

### 5.4. Effect of Receiving FOV Error on Echo Characteristics

**Figure 13:**
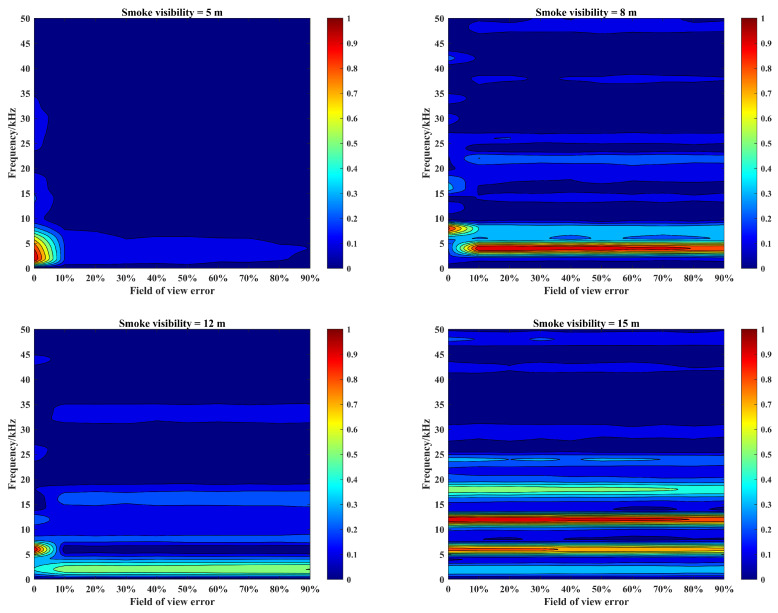
Amplitude–frequency characteristics of beat signals corresponding to divergence angle errors of laser source under different smoke visibility conditions.

Under different smoke visibility conditions, according to the above echo signal simulation process, in order to analyze the effect of photodetector difference on the echo characteristics, the effect of photon receiving FOV error on the target echo characteristics is simulated here. The simulation parameters are still the same as those in Section 3, and the number of FOV error is set to 0–90% of the initial value under the smoke visibility conditions of 5 m, 8 m, 12 m, and 15 m. The amplitude and frequency characteristics of beat signals are shown in Figure 13.

It can be seen that the receiving FOV error will narrow the photon reception area, reducing the reception performance of the photodetector. The impact on the echo characteristics are as follows: (1) The peak migration will appear in the beat signal spectrum. Although the photodetector performance error will cause the peak of the beat signal spectrum to lower frequency band migration, it will not cause a significant reduction in the amplitude. (2) The peak migration of the beat signal spectrum is not affected by the change in smoke visibility, and the threshold at which the peak migration appears always remains stable. Meanwhile, as the FOV error increases, the SNR of echo signals remains basically unchanged. (3) The effect of photodetector performance difference on peak migration is not related to smoke visibility. The peak migration phenomenon of the beat signal spectrum only appears in low-visibility smoke conditions. Thus, the peak shift factor is dominated by frequency change at low visibility, and the peak shift is dominated by amplitude change at high visibility.

### 5.5. Effect Comparison of Error Factors on Echo Characteristics

According to the above analysis results, the laser source emission power error, laser source divergence angle error, and receiving FOV error on the echo characteristics have the peak migration phenomenon of beat signal spectrum. This will obviously cause the reduction in the detection capability of the fuse prototype and the SNR of the echo signal, which makes it difficult to extract the target echo signal effectively and is not beneficial for extracting the distance information of the target.

**Figure 14:**
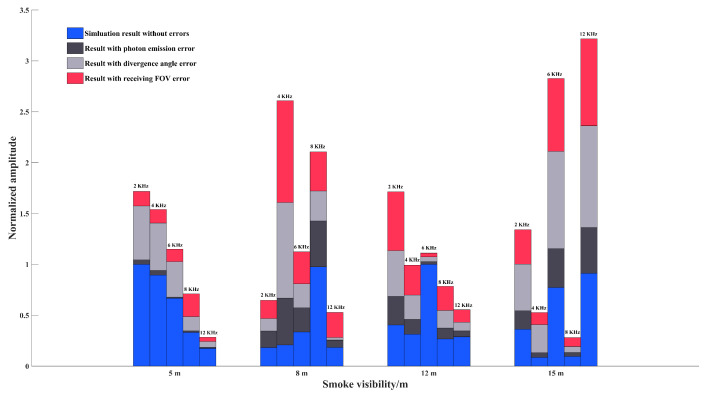
Normalized amplitude result with different types of factors by the means of average.

In order to analyze the importance of the above error factors, it is necessary to compare the effects of the above three types of error factors on the echo characteristics. Therefore, according to the results in Figure 11, Figure 12 and Figure 13, the average amplitude error results of each type of error factor are calculated at different smoke visibilities. The comparison between this average error result and the error-free result is made and the results with 2 KHz, 4 KHz, 6 KHz, 8 KHz, and 12 KHz are shown in Figure 14.

It can be seen that the laser source emission power error has the greatest impact on the echo signal. As the visibility of smoke decreases, the degree of influence of laser divergence angle error on the echo signal will gradually increase. On the contrary, as the visibility of the smoke increases, the influence of the receiving FOV error on the echo signal gradually increases. The reasons for the above situation are as follows: (1) The emission power error directly decreases the number of photons emitted, causing an overall decrease in the amplitude of the echo signal. The other two types of errors do not cause changes in the number of photons emitted. Changes in the transmitting and receiving areas only cause changes in the amplitude of the target echo signal or smoke echo signal alone; thus, they affect the echo signal to a lesser extent. (2) The laser divergence angle is usually much smaller than the received FOV, the effect of the same degree of error on the results depends on the number of particles in the smoke scene. When the number of smoke particles inside the scene is large, a large number of photons will be received by the detector after the back forward scattering of smoke particles. Compared with the receiving FOV error, laser divergence angle error will expand the photon movement area, which will cause a large reduction in the number of photons received by the detector, resulting in a significant change in the amplitude of smoke echo signal. However, when the number of smoke particles inside the scene is small, many photons will be received by the detector after the target reflection. Compared with the laser divergence angle error, FOV error will narrow the scope of the receiving area, which will cause a reduction in the number of photons received by the detector, resulting in a change in the target echo signal amplitude.

## 6. Validity Experiment of Laser Echo Characteristic Simulation Method

Considering the validity and reliability of the Monte-Carlo simulation method, the proposed simulation method is able to obtain correct and reliable results of the echo characteristics in a smoke-free environment. By verifying the validity of the proposed method in smoke environment, the reliability of results for simulating the laser echo characteristics based on the principles of 3D particle system, ray-tracing, and Monte-Carlo principle are demonstrated in this part. The specific method of validation is to compare the correlation and difference between simulated and measured data under the same visibility conditions. In particular, due to the absolute difference between the simulated and measured echo signals under the same visibility conditions, the statistical-based similarity verification of the echo characteristics results in one visibility range is used here, which can reflect to some extent the consistency of the results of different methods in the overall range of smoke visibility and the validity of the echo characteristics simulation methods.

**Figure 15:**
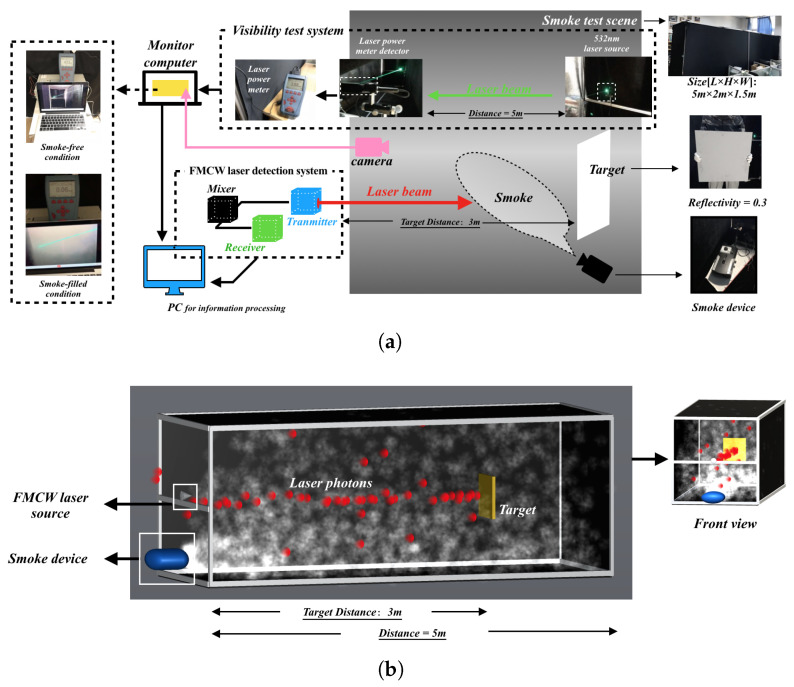
Setup and process of laser prototype test and virtual particle system simulation: (**a**) Laser prototype test; (**b**) virtual particle system simulation.

The effectiveness validation of the proposed simulation method is accomplished by comparing the measured and simulated target echo characteristics under different smoke visibility conditions. According to the above analysis, the specific way is to compare the actual measured data of the detector prototype with the simulated data for the echo characteristics results in the same smoke visibility range. In order to obtain the measured data of the detector prototype, based on the FMCW laser detection process in Figure 1, by building a smoke test environment in the laboratory [37], the test parameters and process are shown in Figure 15a. The modules involved mainly include the following: (1) FMCW laser detection system module—its functions mainly include laser transmission and reception, reception of echo signal, and acquisition of beat frequency signal; (2) Smoke visibility test system module—its function is based on the laser power meter to obtain the visibility in smoke scene [30]; (3) Smoke environment generation module—its function is to generate smoke via the smoke device; (4) Monitoring module—its function is mainly to determine whether the laser spot is completely on the target surface. In order to obtain the simulation data based on the proposed method, according to the principle of 3D particle system simulation in Section 3 and laser echo signal simulation process in Section 5, the smoke device is used as an example to simulate the virtual smoke generation process, and the simulation parameters are set to be the same as the actual test conditions. The smoke test environment built in Unity3D and the simulation process are shown in Figure 15b.

**Figure 16:**
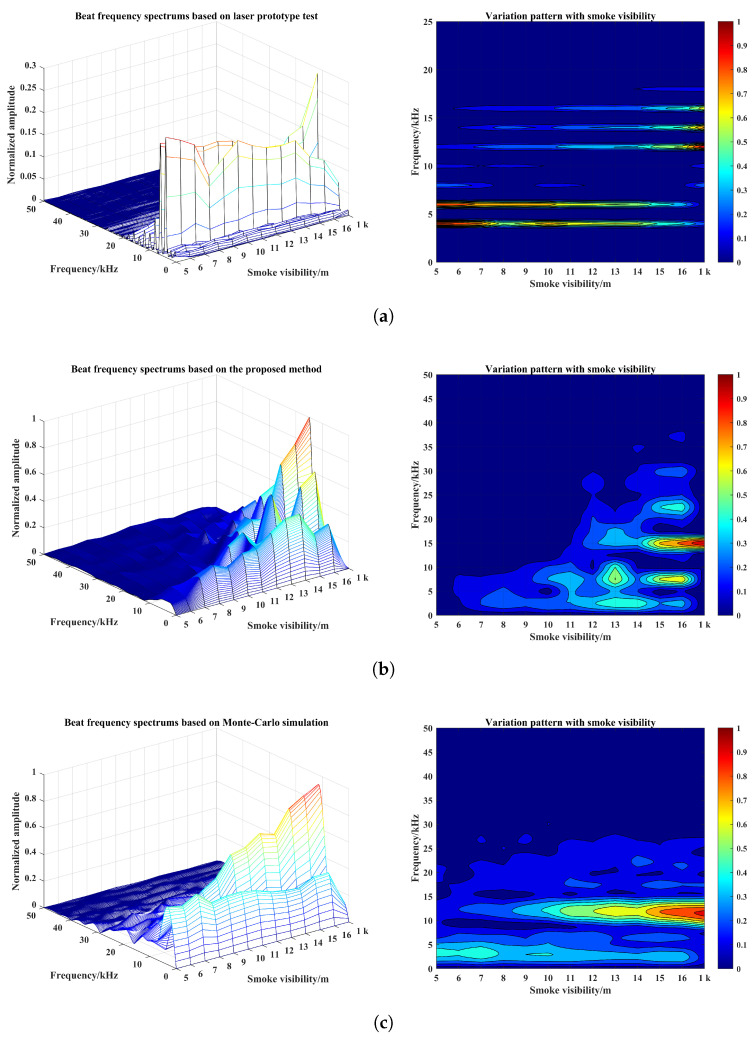
Results of beat signal spectrum and variation pattern with smoke visibility based on three methods: (**a**) laser prototype test, (**b**) proposed method, (**c**) Monte-Carlo simulation.

Meanwhile, in order to show the advantages of the proposed simulation method and differences of echo characteristics results, it is necessary to compare with target echo characteristics results by the Monte-Carlo simulation method, where the simulation setup conditions are consistent with the experimental test conditions. The purpose is to determine whether the echo characteristic results improve the accuracy of real target identification or eliminate the possible false identification of smoke pseudotargets. The Monte-Carlo simulation method proposed by Zhang [15] has been used as the comparison here. By setting the visibility to 1 km in smoke-free environment and a visibility range of 5 m–16 m in the smoke environment, the measured beat signal spectrum and the variation pattern with smoke visibility are shown in Figure 16, with the condition that the simulation setup conditions are consistent with the actual test conditions. The 2D variation pattern is obtained by direct projection of the 3D beat signal spectra onto the Frequency–Smoke visibility plane.

Moreover, in order to show the difference in amplitude between smoke echo and target echo to a certain extent and to reflect the change pattern, the detection distance is calculated based on the frequency corresponding to the extreme of the beat frequency spectrum to distinguish whether the identified target is a smoke pseudotarget or a real target. In this way, it is possible to determine whether the echo characteristics are consistent. From Figure 16a–c, the following can be observed: (1) They all have a positive correlation between smoke visibility and amplitude of the target echo signal, and a basically negative correlation between smoke visibility and amplitude of the smoke echo signal. (2) With the increase in smoke visibility, the beat frequency amplitudes corresponding to target echo signal all have a large growth rate, while the decay rates corresponding to the smoke echo signal are all small. (3) The smoke echoes all have a large value in the low-frequency area of the beat signals. (4) The smoke visibility threshold from the wrong to accurate identification of the target are both present. Therefore, the two simulation methods obtained the same data measurements in the variation pattern of beat frequency with smoke visibility.

Meanwhile, based on Equation (6), which shows the bijection relationship between beat frequency and detection distance, the following can be seen from Figure 16: (1) When the visibility of smoke increases from 5 m to 9 m, the detection distance is not always equal to the actual distance of the target based on the frequency corresponding to the extreme value of the beat frequency spectrum between simulations and actual measurement. (2) When the visibility of smoke increases from 10 m to 14 m, the frequency corresponding to the extreme value of beat frequency spectrum based on the Monte-Carlo simulation is 12 kHz, and the calculated detection distance by Equation (6) is equal to the actual distance of the target; the real target can be identified at this time. However, the proposed simulation method and measured data of beat frequency corresponding to spectrum extreme value is not equal to 12 kHz; these two methods can only identify the smoke pseudotarget, and cannot identify the real target. (3) When the visibility of smoke increases to 15 m, the actual distance of the target can be calculated directly based on the frequency corresponding to the extreme value point of the spectrum of simulation and experiment, and the real targets can all be identified accurately at this time.

In summary, under a certain smoke visibility range, whether the real target can be identified or not, the proposed simulation method is not only consistent with the target echo characteristics results obtained from the real test, also more consistent than the echo characteristics results of the basic Monte-Carlo method, which indicates the validity and reliability of the proposed echo characteristics simulation method. In addition, since the virtual simulation conditions are not completely consistent with the experimental test conditions, the SNR of the beat signal and the detection distance results in the local interval obtained from the simulation will always have some differences. However, the correctness of the above conclusions is not affected. By increasing the accuracy of the smoke simulation, more precise conclusions can be extracted from the above findings.

**Figure 17:**
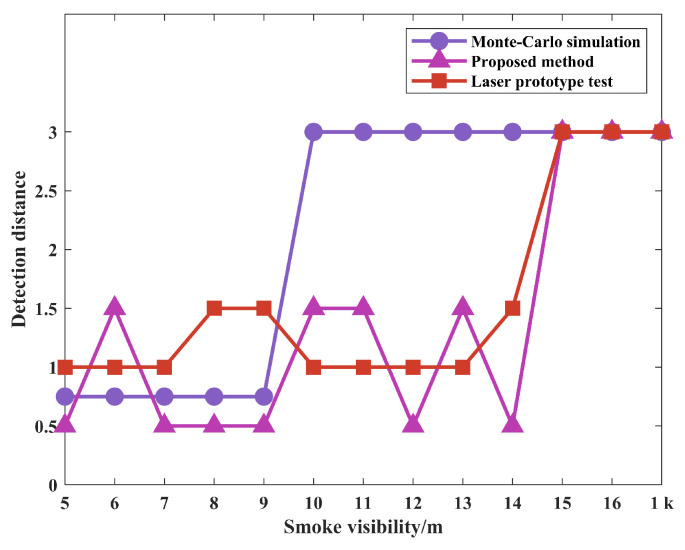
Detection distance results.

Moreover, in order to objectively represent the similarity and difference of the echo characteristics results between the two simulation methods and the actual results measured by laser prototype—especially the detection distance results, which are shown in Figure 17—the following evaluation indicators are used here for characterization: The correlation degree of distance results ρ based on the correlation coefficient, the difference of distance results *e* based on the mean square error, the false alarm rate fc based on the target misidentification, and the accuracy rate ac based on the real target identification. The specific calculation formulas are shown in Equations (18)–(21), respectively, and the calculation results are shown in Table 1.

For the correlation of detection results, the larger the value of ρ, the greater the correlation between simulation and real measurement; the smaller the value of *e*, the greater the correlation between simulation and real measurement. For the accurate identification of the real target, a smaller value of fc indicates that the difference in target echo characteristics between simulation and measurement is smaller, and the effectiveness of the simulation method is better. A larger value of ac means that the difference between simulation and measurement is smaller, and the validity of the simulation method is better at this time. As can be seen in Table 1, compared with the basic Monte-Carlo simulation method, the constructed optimization and simulation method based on the principle of 3D particle system, ray tracing, and Monte-Carlo method has greater correlation with the measured results. At the same time, the false alarm rate can be reduced under the condition of consistent correctness, and the effectiveness of echo characteristic simulation method is also better. In addition, ac of proposed simulation method is not improved because the smoke environments in both methods are obtained based on smoke visibility simulation only. The indicator can certainly be improved by enhancing the simulation accuracy of the smoke environment.

(18) $$\rho =\frac{\sum_{V=5m}^{max}\left(R_{simulation}\left(V\right)-{\bar{R}}_{simulation}\right)\left(R_{lasertest}\left(V\right)-{\bar{R}}_{lasertest}\right)}{\sqrt{\sum_{V=5m}^{max}{\left(R_{simulation}\left(V\right)-{\bar{R}}_{simulation}\right)}^{2}}\sqrt{\sum_{V=5m}^{max}{\left(R_{lasertest}\left(V\right)-{\bar{R}}_{lasertest}\right)}^{2}}},$$

(19) $$e=\frac{1}{13}\sum_{V=5m}^{max}{\left(R_{simulation}\left(V\right)-R_{lasertest}\right)}^{2},$$

(20) $$fc=\frac{Number_{Misidentifications}}{Number_{Totalexperiments}},$$

(21) $$ac=\frac{Number_{AccurateIdentifications}}{Number_{Totalexperiments}},$$

## 7. Conclusions

In this paper, based on particle dynamic collision, reverse ray-tracing, and FMCW laser echo signal model, a new simulation model of FMCW laser transmission is built using the professional particle system of Unity3D. In order to analyze and compare the effects of performance variation on the echo characteristics, such as laser source transmit power error, laser source divergence angle error, and received field of view error, the effect of laser fuse detector component difference on the target echo characteristics is studied by simulation in a smoke environment. It is found that all three types of errors cause the peak migration phenomenon of the beat frequency spectrum: (1) Only the peak migration phenomenon of the spectrum under laser source transmit power error is affected by the smoke visibility; (2) only the laser source divergence angle error causes the peak migration phenomenon of the spectrum in high visibility; (3) only the effect of received field-of-view error on the peak migration phenomenon is independent of the smoke visibility. The results show that the SNR of echo signals is not only related to the smoke visibility, the performance variation of detector components also seriously reduces the SNR by peak migration. In particular, based on the analysis of laser-source-emitted power error, it is the most disadvantageous factor for the accurate identification of the target.

The effectiveness of the proposed simulation method is verified by comparing the results of target echo characteristics obtained from the experiments and simulations. For the identification of real targets, the proposed simulation method has the same echo characteristic results as real measurement data by the prototype. It is reflected the reliability of simulation results in the paper. Compared with the basic Monte-Carlo method, the false alarm rate of laser echo characteristics simulation results is reduced by 38%, while maintaining the same target identification accuracy. Moreover, based on the significant effect of laser source emitted power error, it is indicated that laser fuse can be considered for multiple laser emission structures for improving the target detection capability when the laser source performance is likely to lose more than 30%. So, based on the simulation method and results, the research on optimization of internal performance parameters and external structure design of FMCW laser fuse antismoke interference should be further studied.

## Figures and Tables

**Table 1:** Results of evaluation indicators between Monte-Carlo simulation and proposed method.

Method	ρ	*e*	fc	ac
Monte-Carlo simulation	0.73	1.50	0.38	0.23
Proposed method	0.81	0.37	0.00	0.23

## Data Availability

Not applicable.
